# Principal Causes of Constitutional Derangement in First Dentition

**Published:** 1874-08

**Authors:** J. Hardman


### ARTICLE IV.

### *Principal Cause of Constitutional Derangement in First Dentition.*

BY J. HARDMAN.

Read before the Iowa State Dental Society, May, 1874.

This subject has received the attention and investigation of eminent medical men for centuries, and many are the views from the pens of learning in regard to the laws governing and the circumstances attending this process, at this critical period of the child's life.

The enigma—the mystery surrounding this subject of difficult first dentition, has been a source of great aggravation to the enquiring mind, exhibited in the great extremities of its effects. At one time the beautiful and expectant pearl has come so normally and so gracefully that the tranquility and health of the little pet has not in the least been disturbed. When, again, its advance and eruption is announced by symptoms of suffering and disaster that not only excite anxiety among parents and friends, but alas too often ends the tender life of the little sufferer.

Many are the affections that difficult dentition does often induce. Congestions, inflammations, with their attending consequences, attacking any of the vital organisms, producing cholera infantum, dysentery, meningitis, convulsions, etc., in all their direst severity. Able authors have, in our text-books, advanced theories to account for this wonderful phenomenon; have given what they regard as the cause of such extensive and severe effects, arising apparently from so insignificant a change of structure. And we read and re-read, weigh and consider, and we find ourselves not satisfied with the arguments advanced and many of the conclusions arrived at.

We ask ourself if the physico-vital forces engaged in building up the tooth, cell by cell, and at the same time cell by cell is dissolved and carried away from the surrounding alveolus to fully prepare and maintain ample space without creating the least visible derangement, not only in the immediate parts, but also in the general system. What is it that can so disturb the vital harmony at the time the tooth is just about erupting, and in many cases partially through?

To supply somewhat of a plausible reply to this perplexing question this feeble paper has its humble aim, establishing if possible, a more rational and effective mode of treatment. Permit us then, while we ask your respectful attention, to first glance briefly at the usually accepted theory. Secondly, present what we regard the true theory. And thirdly, the rational local treatment as indicated from this stand point.

We will glance at the anatomical structure, and the physiology of the trigemini, and its co-workers the sympatheticus major.

The fifth pair of nerves rise low in the base of the brain, and with faciculi or filaments of origin descending down *via*, the pons varoli and medulla oblongata, making it a spinal as well as an incephalic nerve,—bearing in mind that it mostly supplies with its branches all the important parts of the mouth. The great sympathetic nerve in connection with the trigemini, furnishes extensive influence by its numerous ganglia plexus, anastomosis, etc. This latter nerve is as it were the connecting bond of the entire system of nerves, exerting and transmitting wonderful effect, both of voluntary and involuntary force. This nerve, as it descends, sends off, externally and internally, branches to every portion of the body, contributing evidently not only to the sensibility and motion but also to special function in most of the vital organs. These then, the trigemini and the great sympathetic, are not only anatomically connected, but exhibit great influence over the general system. It is, therefore, when we contemplate this universal and intimate connection of the nervous system that we, to some degree can comprehend some of the mysterious phenomena where a local irritant in one part or organ may establish the most marked effects in another part or organ. Thus it has been observed where the pain in the knee was the result of disease in the hip joint. Where, in the adult, the dental surgeon so frequently meets, in the so-called neuralgia, the seat of pain and distress quite remote from the seat of disease. So in the infant; irritation in the alveoli through the media of impression made upon and through these great nerves, the stomach, bowels, liver, kidneys, lungs, brain, etc., are often the parts upon which the disastrous force is centred, thus causing impaired digestion, diarrhœa, spasms, coughs, convulsions, cephlic congestions and death.

That these dire effects occur from first dentition has long been observed; that the subject is intimately connected with the calling and duties of the dental practitioner is quite obvious. It is then a question of vital importance to the dentist to know the true cause of constitutional derangement in first dentition.

Dr. Delabarre thought it arises from a defection in the contraction of the investing dentinal capsule: while the lamented Dr. Harris more philosophically thought it may depend upon the pulp being pressed upon, this being caused by the unyielding gums after the osseous opening of the socket has relieved the point of the tooth. The former of these is a vain theory that has no commendable quality; the latter more plausible, but certainly defective in ascribing the pressure upon the nerve as arising from the resistance offered by overlying gums or soft parts. Drs. Meig, Wood, Bond, Condie, Dunglison and a host of others impute also to the resistance offered by the gums as the seat and origin of the mischief. We wish, notwithstanding this great weight of talent before us, to be understood as placing no estimate upon the theory that the resistance from the gums contribute in any comparative degree towards causing the constitutional trouble. But we do believe, most confidently, the cause to arise from pressure upon the large and very vital pulp. The question at issue, as mostly claiming our attention, is from whence comes this irritating pressure? And why the mischievous effects from it.

We maintain that the resistance offered from the gums is of but small moment. That the true cause arises from pressure upon the nerve or pulp is certain. And that this pressure comes from an indirect way, namely: by the growth of the lower or root part of the tooth being too fast, so to speak, when compared to the removal of the osseous covering over its crown, thus holding it back, and it thereby pressing unduly and irritating the pulp.

We will here, then, in order the more fully to present our views, note what may be regarded as two axioms, viz:

1. Normal pressure promotes absorption of osseous tissue.
2. Too much, or undue pressure will, by inducing irritation, arrest absorption. It has long since been admitted, and backed by respectable proof, that a given amount of pressure being exerted will, by healthy, physiological action, stimulate the removal of osseous structure. And of this principle the dentist takes advantage in the treatment of irregular teeth, even in the mature jaw. But where a pressure becomes excessive, instead of a stimulation to activity, the normal function of the membrane compressed is by impediment of circulation, etc., irritated and inflamed, in many cases totally arresting action, save that proper to pathological law—namely, disease.

When we consider that the growth of the deciduous teeth is mainly by deposit of cell by cell, and the filling in of lime salts particle by particle, from the crown towards the apex; and further, that the pulp or the nerve at this period forms a large portion of the contents of the dentinal capsule, and is of a highly vital organization; and still further, that the normal growth of this portion of the tooth does produce an upward pressure upon and against the capsular covering of the tooth, or lining of the bone; and that through this means in some way the osseous structure is dissolved and carried away. This process is usually normal before the first opening occurs in the bony casement, as there is seldom evidence of trouble up to this time.

It should be born in mind that the greatest tendency to constitutional disturbance arises from the eruption of the cuspid teeth. These being the most pointed in form we might readily be induced to think they should be most favorable to spontaneous eruption. It is, however, reasonable to conclude from observing the effects, and weighing the real principles of action, that the conical form is no advantage, but instead thereof, the reverse. The point may be brought through the osseous casement, and, in some instances, also through the soft covering of the alveolus, yet the acme of trouble not reached.

We shall endeavor here to show the bearing that the process of eruption in this peculiar form of teeth has upon this subject. We maintained that this phenomenon is owing to undue pressure upon the pulp, and not upon the tissues in advance of the cutting crown surface. This, then, is our theory, taking a cuspid as a case for illustration. The point is readily felt by the lancet in the hand of the surgeon; but his crucial incisions in the soft tissues have but little effect upon the attending constitutional symptoms, and why? To answer let us seek whence this undue pressure.

The point of the tooth having penetrated the bony surface, a large amount of resistance proper from below is taken off, hence allowing a greatly increased force being exerted upon the borders of the socket opening, and this force of pressure being too great is an irritant upon the portion of the capsule surrounding this opening, creating inflammation in it and thus arresting absorption. Holding the tooth, in other words, immovably fixed. While thus stationary the growth of the tooth below still going on, must, it is obvious in its effect, produce indirectly pressure upon the pulp. And this is the whole mystery, undue pressure augmented by a portion of resisting surface already removed from over the tooth, and by irritation and inflammation arresting absorption, fixing the tooth in the socket, while the steadily increasing and elongated tooth, indirectly but surely, producing the pernicious pressure upon the pulp; and this in turn becoming by this pressure irritated and inflamed, producing in remote organs, through the intimate nervous union before referred to, the extensive sympathetic disturbance so often met.

It would seem from this illustration that we regard this abnormal change as much more liable in the cuspid teeth, as taking place at this critical time, and that the other teeth are to some extent exempt. This, we do think, is very much the fact; and account for it in this way. These conical teeth have their largest diameter near the middle or base of the crowns; thus, at the time of penetration of the alveolus by the point, and the consequent removal of an equivalent amount of resistance, a much larger surface of the capsule is left to receive the augmented and irritating pressure. Whereas, in the more perpendicular crowns, with larger surfaces, being at once uncovered, leaves but little surface of membrane exposed to this over pressure, and consequent irritation. And we appeal to the experienced practitioner, if it is not usually the capsule in which the main constitutional disturbance is found.

From the drift of the description, it will be noticed that we dwelt upon the finding and fixing of the true local cause and its pathology; but leaving the mysterious philosophy of sympathetic action and phenomena mainly unnoticed. It will, however, be born in mind, that we aimed to point out the cause and not the effect of this constitutional derangement. We do, however, regard it as befitting and right to close with a word as to the mode of local treatment in first and difficult dentition.

It follows from this view that we have taken, that the condition of the soft parts over the tooth is of small moment, save in diagnosis. But that the point to refer our attack in treatment is to relieve the tooth from its strangulation in the bony casement, and thus materially, effectually relieve the pulp from the undue pressure. To do this we would suggest that with a spear shaped chisel, or stiff lancet blade, the portion of the alveölus overlying and surrounding the impinged portion of the tooth be heroically broken up. Anything short of this will not meet the demands of the case. The only plausible good the mere incising of the soft parts may have, must be in the reduction of engorgement of the gums through the loss of blood, which must, of course, amount to very little.

As testimony of this radical practice, we can say that we have seen in cases coming under our immediate observations and care, the very best results. But we would insist here, that to be effective, the surgeon should not hesitate to remove or break up the bony casement freely down to or nearly to the largest diameter of the crown.—*Dental Register*.

